# SHMT2 regulates esophageal cancer cell progression and immune Escape by mediating m6A modification of c-myc

**DOI:** 10.1186/s13578-023-01148-7

**Published:** 2023-11-06

**Authors:** Zhe Qiao, Yu Li, Yao Cheng, Shaomin Li, Shiyuan Liu

**Affiliations:** Department of Thoracic Surgery, The Second Affiliated Hospital of Xi’an Jiaotong University, No. 157, West 5th Road, 710004 Xi’an, Shaanxi China

**Keywords:** SHMT2, EC, m6A modification, c-myc, One carbon metabolism

## Abstract

**Background:**

In recent years, the role of altered cellular metabolism in tumor progression has attracted widespread attention. Related metabolic enzymes have also been considered as potential cancer therapeutic targets. Serine hydroxymethyltransferase 2 (SHMT2) has been reported to be upregulated in several cancers and associated with poor prognosis. However, there are few studies of SHMT2 in esophageal cancer (EC), and the related functions and mechanisms also need to be further explored.

**Methods:**

In this study, we first analyzed SHMT2 expression in EC by online database and clinical samples. Then, the biological functions of SHMT2 in EC were investigated by cell and animal experiments. The intracellular m6A methylation modification levels were also evaluated by MeRIP. Linked genes and mechanisms of SHMT2 were analyzed by bioinformatics and rescue experiments.

**Results:**

We found that SHMT2 expression was abnormally upregulated in EC and associated with poor prognosis. Functionally, SHMT2 silencing suppressed c-myc expression in an m6A-dependent manner, thereby blocking the proliferation, migration, invasion and immune escape abilities of EC cells. Mechanistically, SHMT2 encouraged the accumulation of methyl donor SAM through a one-carbon metabolic network, thereby regulating the m6A modification and stability of c-myc mRNA in a METTL3/FTO/ALKBH5/IGF2BP2-dependent way. In vivo animal experiments also demonstrated that SHMT2 mediated MYC expression by m6A-methylation modification, thus boosting EC tumorigenesis.

**Conclusion:**

In conclusion, our data illustrated that SHMT2 regulated malignant progression and immune escape of EC cell through c-myc m6A modification. These revealed mechanisms related to SHMT2 in EC and maybe offer promise for the development of new therapeutic approaches.

**Supplementary Information:**

The online version contains supplementary material available at 10.1186/s13578-023-01148-7.

## Introduction

Esophageal cancer (EC) is one of the most lethal malignancies in the world and also the most aggressive among gastrointestinal malignancies. The incidence of EC continues to rise and is often diagnosed at an advanced stage due to the lack of early clinical signs, resulting in poor prognosis and low survival [1]. In recent years, despite improvements in the management and treatment of patients with EC, overall outcomes remain poor, with 5-year survival rates only approximately 15–25% [2]. Early-stage patients can be successfully treated by endoscopic resection, but patients with advanced EC cannot be cured by surgery alone, and a multidisciplinary approach is needed to improve outcomes [3]. Therefore, there is a need for an in-depth study of the biological mechanisms of EC development to find new therapy targets, ultimately improving patient prognosis.

Studies have shown that cancer cells re-edit metabolic processes, such as glycolysis, tricarboxylic acid cycle, and glutamine metabolism, in order to maintain rapid cell proliferation during tumor development, thereby maintaining cellular biosynthesis, energy production, and redox homeostasis [4]. This metabolic reprogramming strengthens growth and proliferation of tumor cells by involving altered expression and functions of metabolism-related genes [5]. Alterations of metabolic pathways have also become a new direction and hotspot in tumor research. Metabolic disorders also underlie the development of the upper gastrointestinal malignancies. In addition to dysregulation in glucose metabolism (as shown by the well-known Warburg effect), amino acid, lipid and nucleotide metabolism are observed *in vitro and in vivo* as well [6]. Several studies have demonstrated that upper gastrointestinal cancers have significant changes in nucleotide metabolites. Nucleotide synthesis and metabolism are required for adequate energy production and are essential for cancer cell proliferation and differentiation [7–9]. Therefore, studying and revealing the key metabolic pathways and targets associated with EC progression could provide insight of cancer etiology, leading to the development of novel preventive approaches and therapeutic targets.

A previous study revealed metabolic shifts in EC, including glycine, serine and threonine metabolism and glycerolipid metabolism. Furthermore, glycine levels were positively correlated with disease progression [10]. Metabolic enzymes not only connect and regulate complex metabolic reactions in the biological metabolic networks, but are also considered as potential anti-cancer targets [11]. Serine hydroxymethyltransferase 2 (SHMT2) is a noteworthy metabolic enzyme that is a key metabolic enzyme for the conversion of serine to glycine. SHMT2 is mainly distributed in mitochondria, containing 10 introns and 11 exons with multiple consensus motifs for regulatory proteins in the 5’ end promoter region [12]. SHMT2 has been reported to be associated with the progression of various tumor cells, such as cell proliferation, migration, invasion and apoptosis. Dohoon et al. revealed that SHMT2 scavenged glycine during ischemia and affected pyruvate kinase (PKM2) activity, thereby inducing cell proliferation in human glioblastoma multiforme [13]. Liu et al. illustrated that a positive feedback loop formed by β-catenin and lysine residues of SHMT2 pushed the process of colorectal carcinogenesis and development [14]. Zhang et al. confirmed that SHMT2 was highly expressed in bladder cancer and correlated with overall survival. Cell proliferation was increased and apoptosis was decreased when SHMT2 is overexpressed by regulating the STAT3 signaling pathway [15]. However, its function and potential mechanisms in regulating EC progression and metabolic reprogramming remain largely unknown.

In the current study, we revealed that SHMT2 was aberrantly augmented and correlated with poor prognosis in EC through analyze of database analysis and clinical data. Further bioinformatic analysis identified that SHMT2 was most linked with MYC. Next, we focused on the function and mechanism of SHMT2 in EC by *in vitro and in vivo* experiments. We demonstrated that SHMT2 regulated the malignant progression and immune escape of EC cell through c-myc m6A modification. By interpreting the function and mechanism of SHMT2, it may provide promising biomarkers of targeting metabolic reprogramming for the EC novel treatment.

## Materials and methods

### Clinical samples

In this study, tumor tissues and adjacent normal paracancerous tissues were collected from 30 EC patients with EC treated at the Thoracic Surgery Department of the Second Affiliated Hospital of Xi’an Jiaotong University. According to the TNM stage, there were 15 patients in I-II stage and 15 patients in III-V stage. All patients had not undergone any interventions such as radiotherapy prior to surgical resection tissues. All isolated tissues were immediately snap frozen by liquid nitrogen in lyophilized tubes and then transferred to -80 °C refrigerator for storage. In addition, written informed consent was obtained from all patients prior to study begin. The study was also approved by the Second Affiliated Hospital of Xi’an Jiaotong University Ethics Committee and was conducted in accordance with the Declaration of Helsinki.

### Cell culture

The human normal esophageal epithelial cell line HEEC and four cancer cell lines (TE-1, ECA109, KYSE150, TE-10) were purchased from the cell bank of the Chinese Academy of Sciences. Cells were cultured using RPMI-1640 medium (Corning) containing 10% FBS (Gibco) and an incubator at 37 °C with 5% CO_2_. When the cell density reached 80%, cell passaging was performed.

### RT-qPCR

Total DNA was isolated from cells or tissues using the RNeasy kit (Qiagen). The quality and purity of RNA was checked using a NanoDrop® ND-1000 spectrophotometer (Thermal Fisher). For RT-qPCR, RNA was reverse transcribed to cDNA using the PrimeScript RT kit (Takara). All samples were subjected to PCR reactions using the Hieff® qPCR SYBR Green Master Mix (Yeasen). The relative quantification of mRNA was calculated using method 2^−ΔΔCt^ and normalized to GAPDH. Primer sequences were as follows:

**Table:** 

	Forward primer	Reverse primer
SHMT2	CCCTTCTGCAACCTCACGAC	CCCTTCTGCAACCTCACGAC
METTL3	TTGTCTCCAACCTTCCGTAGT	CCAGATCAGAGAGGTGGTGTAG
METTL14	AGTGCCGACAGCATTGGTG	GGAGCAGAGGTATCATAGGAAGC
WTAP	CTTCCCAAGAAGGTTCGATTGA	TCAGACTCTCTTAGGCCAGTTAC
FTO	ACTTGGCTCCCTTATCTGACC 3	TGTGCAGTGTGAGAAAGGCTT3
ALKBH5	CGGCGAAGGCTACACTTACG	CCACCAGCTTTTGGATCACCA
c-myc	CGTCTCCACACATCAGCACAA	TCTTGGCAGCAGGATAGTCCTT
GAPDH	ACAACTTTGGTATCGTGGAAGG	GCCATCACGCCACAGTTTC

### Western blot

Cells or tissues were lysed by RIPA buffer (Beyotime). Protein concentration was measured using the BCA Protein Assay Kit (Beyotime). Equal amounts of total protein were separated using 10% SDS-PAEG (Beyotime) and transferred to PVDF membranes (Millipore). The membrane was then closed with 5% BSA (Beyotime) for 1 h at room temperature. The membranes were then reacted with primary antibodies against SHMT2 and GAPDH (Abcam) at 4 °C overnight. The next day, the membranes were incubated in HRP-coupled secondary antibody (Abcam) for 1 h at room temperature. Finally, protein bands are visualized using chemiluminescent substrate (Millipore) and quantified using ImageJ.

### Cell transfection

For transient transfection: siRNA and pcDNA plasmids were designed and synthesized by Guangzhou RiboBio. Lipofectamine 3000 (Invitrogen) was applied to transfect the cells with siRNA or plasmids according to the manufacturer’s protocol. After 48 h transfection, RT-qPCR was employed to evaluate the transfection efficiency of the target gene.

For stable transfection: shRNA lentiviral packaging plasmid vector targeting SHMT2 was purchased from Guangzhou RiboBio, and sh-NC was served as control. Cells were transfected with lentiviral packaging plasmids. The transfection efficiency was improved using polybrene (6 ug/ml). Fresh complete medium was replaced after 8 h of infection. Subsequently, positive cells were screened by puromycin (200 ug/ml) after 24 h and the medium was changed regularly for the next 2 weeks. Finally, RT-qPCR was conducted to analyze stabilize transfection efficiency of the target genes.

### MTT assay

Cells were inoculated in 96-well plates and 20 µl of MTT (Sigma) solution was added to each well at 0, 24, 48 and 72 h. After 4 h of culture, the medium was discarded and 150 µl of DMSO (Solarbio) was augmented. Subsequently, a shaker was employed to shake at low speed for 10 min to fully dissolve the purple crystals. Finally, the absorbance at 490 nm was measured using a microplate reader to assess the proliferation capacity of the cells.

### Colony formation assay

Grouped cells in logarithmic growth phase (2.5×10^4^) were added to 6-well plates. After 14 days, when clonal clusters of cells were observed, the culture was stopped. clonal clusters were washed with PBS and fixed with 4% paraformaldehyde (Beyotime) for 15 min. Crystalline violet was then applied to stain. Finally, a microscope was employed for photographing and counting.

### Transwell assay

For cell migration, cells were resuspended in serum-free RPMI-1640. Then 100 µl cell suspension was inoculated into the upper chamber of a 24-well Transwell plate (8 μm pore size, BD Biosciences). Next, 600 µl of complete medium was added to the lower chamber. After 48 h, migrated cells in the lower chamber was fixed with 4% paraformaldehyde for 15 min and stained with 0.1% crystalline violet for 10 min. Finally, the cells were photographed and counted using a microscope. For cell invasion, transwell chambers were treated with matrix gel prior to cell inoculation, and the rest of the procedure was the same as for the cell migration assay.

### Reagent kit assay

According to the producer’s directions, LDH activity, ATP levels, glycine production or SAM levels were measured using the Lactate Dehydrogenase Assay Kit (Beyotime), ATP Assay Kit (Beyotime), Human Glycine Assay Kit (Fantaibio) or Human S-Adenosyl Methionine (SAM) Assay Kit (Fantaibio), respectively.

### m6A level assay

According to the producer’s directions, the m6A RNA Methylation Quantification Kit (Epigentek) was conducted to measure m6A methylation levels. Briefly, m6A RNA capture was performed using RNA sample reacted with m6A antibody (Sigma) at room temperature. The signal at 450 nm was then detected using a microplate reader and m6A levels were quantified based on absorbance values.

### MeRIP

The Magna MeRIP m 6 A kit (Millipore) was applied for MeRIP. firstly, m6A antibody or negative control IgG (sigma) was incubated with magnetic beads at 4 °C for 3 h. RNA fragmentation was then performed using RNA fragmentation buffer. Subsequently, 10% of the RNA was stored as ‘Input’ in order to serve as a positive control after subsequent RNA purification. The remaining supernatant was incubated with antibody-containing magnetic beads in MeRIP immunoprecipitation buffer at 4 °C overnight. After the magnetic beads were fully adsorbed, MeRIP elution buffer was added and washed 5 times. Later, the immunomagnetic bead precipitates were reacted with elution buffer containing RNase inhibitors at 4 °C for 1 h. Finally, RNA was purified using the RNA purification kit. Eventually, m6A enrichment was analyzed by qPCR with specific primers, and quantification was normalized to input.

### RIP

RIP was performed using the Magna RIPTM RNA binding protein immunoprecipitation kit (Millipore). Cells were washed with ice-cold PBS and lysed in complete RNA lysis buffer. Cell lysates were then incubated with antibody-bound protein A agarose beads for 3 h at 4 °C (IgG as a negative control). The samples were then reacted with Proteinase K and the immunoprecipitated RNA was also isolated. In the end, RT-qPCR was conducted to assay the enrichment of related mRNAs.

### RNA pull down

Cell lysates were incubated with biotin-labeled MYC probes (RiboBio) at 4 °C overnight after incubation. Next, streptavidin magnetic beads (Invitrogen) were incubated with the reaction mixture at 4 °C for 4 h. The beads were then washed with pull-down buffer. Subsequently, the RNA-protein binding mixture was boiled in SDS buffer for western blotting analysis.

### mRNA stability assay

Cells were inoculated in 12-well plates overnight and then treated with 5 µg/mL actinomycin D (MedChemExpress) at 0, 3, 6 and 9 h. Total RNA was then isolated and the results were analyzed by RT-qPCR.

### Dual luciferase reporter gene assay

We cloned the wild-type sequence containing the m6A binding site of c-myc or five mutant-type sequence of m6A binding site (site1-5) into the pmirGLO reporter vector (Promega), named WT, Mut1-5, respectively. Then, the above luciferase vectors were co-transfected with sh-METTL3, sh-FTO or sh-ALKBH5 cells using Lipofectamine 3000. After 48 h, luciferase activity was assessed using a dual luciferase reporter assay system (Promega) based on the producer’s directions.

### Immunofluorescence in situ hybridization analysis

RNA fluorescence in situ hybridization was performed using an RNA probe specific for c-MYC according to the manufacturer’s instructions. The fluorescence in situ hybridization kit and Alexa Fluor 546 labeled c-MYC probe were designed and purchased by RiboBio (Guangzhou, China). Cell crawls were placed in 24-well plates and pretreated with 200 µL of 5% gelatin per well for at least 30 min. Each well was then inoculated with 4 ×10^4^ cells. After grouping for the appropriate time, the cells were fixed using 4% paraformaldehyde. The cells were then permeabilized with 0.5% Triton X-100 working solution for 20 min. After prehybridization, hybridization was performed with the c-MYC probe in hybridization buffer at 55 °C overnight. Subsequently, the wells were closed with 0.5% BSA for 1 h. Next, the cells were incubated overnight at 4 °C using METTL3, ALKBH5 and FTO antibodies. The next day, the primary antibody was recovered, followed by reacting with secondary antibody coupled with Alexa Fluor 488 at room temperature for 2 h. After recovery of the secondary antibody, the cells were stained with DAPI for 5 min. Anti-quenching agent was dripped onto the slides, followed by sealing the crawls with nail polish and air-drying away from light. Finally, they were observed under confocal microscope and photographed.

### Nude mice xenograft Tumor experiment

Four-week-old male nude mice were purchased from The Second Affiliated Hospital of Xi’an Jiaotong University. The mice were acclimatized and fed for one week. Then, they were randomly divided into two groups: sh-NC group and sh-SHMT2 group. Cells transfected with sh-NC or sh-SHMT2 were subsequently cultured routinely. Next, cells with logarithmic growth phase (1 ×10^6^) were taken and injected to the right axilla of nude mice. After subcutaneous inoculation, the mice were observed for their mental status, activity, and tumor formation. The tumor volume was monitored every 4 days, and the mice were euthanized after 28 days. Tumor tissues were separated and weighed from nude mice. A portion of the dissected tumor tissue was fixed overnight in 4% paraformaldehyde, embedded in paraffin blocks and sectioned. The tissue sections were stained with H&E or immunohistochemistry (IHC) according to standard procedures. The remaining tumor tissues were stored at -80 °C for subsequent analysis. All animal experimental procedures were approved by the Second Affiliated Hospital of Xi’an Jiaotong University Hospital Animal Experimentation Ethics Committee and were performed in accordance with its animal welfare principles.

### Statistical analysis

All data in this study were statistically analyzed using GraphPad Prism 8.0 software. Each experiment was independently repeated three times or more. Final results were expressed as mean ± standard deviation. When P values was less than 0.05, the discrepancies were served as significant.

## Results

### SHMT2 is obviously augmented and linked to poor prognosis in EC

**Fig. 1:**
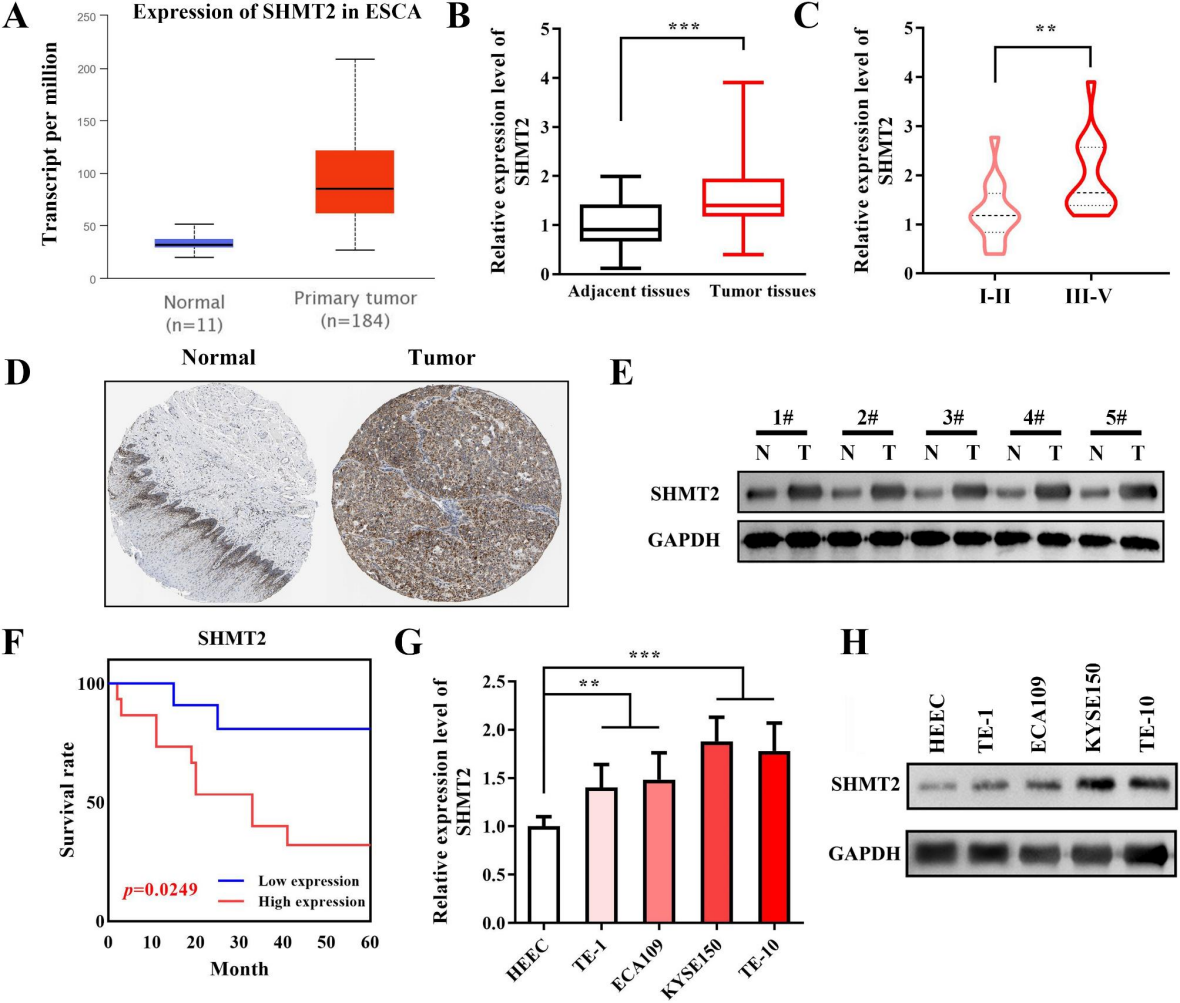
SHMT2 expression and prognosis in EC. **A**: TCGA was applied to analyze SHMT2 expression in EC; **B**: RT-qPCR was employed to detect SHMT2 expression in normal and EC tissues; **C**: SHMT2 expression in different stages of EC patients; **D**: HPA database was conducted to validate SHMT2 expression in EC tissues and normal tissues; **E**: SHMT2 protein expression in EC tissues and paracancerous tissues was assayed by western blot; **F**: relationship between SHMT2 expression and prognosis was evaluated by Kaplan-Meier curve; **G**: SHMT2 mRNA expression in human normal esophageal epithelial cell lines and cancer cell lines; H: SHMT2 protein expression in human normal esophageal epithelial cell lines and cancer cell lines. *P < 0.05; **P < 0.01; ***P < 0.001

Accumulating evidence suggests that SHMT2, a key enzyme in one-carbon unit metabolism, is remarkably associated with malignant progression of several human cancers. Previous studies revealed that SHMT2 expression is significantly induced in gastrointestinal tumors and caused the malignant progression and poor prognosis, including EC [16]. However, how SHMT2 regulates EC progression and the underlying mechanisms remain unclear. In our study, we first analyzed SHMT2 expression in EC patients from the TCGA database. SHMT2 expression was clearly elevated in EC compared to normal tissue (Fig. 1A). Next, we verified SHMT2 expression in the collected clinical tissue samples. SHMT2 expression was notably enhanced in tumor tissues (Fig. 1B). In addition, we revealed that SHMT2 was obviously higher in tumor tissues with high TNM stage (III-V) than those with low stage (I-II) (Fig. 1C). Then, the data of the Human Protein Atlas (HPA) database showed that EC tissues presented higher expression of SHMT2 protein than normal tissues (Fig. 1D). We also assayed the protein expression of SHMT2 in clinical tissue samples. Undoubtedly, the protein level of SHMT2 was visibly increased in tumor tissues (Fig. 1E). What’s more, we further explored whether the high expression of SHMT2 relevant to the poor prognosis in EC. Therefore, we analyzed the meaningful of SHMT2 expression in the survival prognosis of EC patients by Kaplan-Meier. Our results showed that high SHMT2 expression was evidently linked to the decreased patient survival (p = 0.0249, Fig. 1F). In addition to clinical samples, we also examined SHMT2 expression in multiple EC cell lines. Compared to the human normal esophageal epithelial cell line HEEC, SHMT2 expression were sharply heightened (Fig. 1G-H) in all four EC cell lines (TE-1, ECA109, KYSE150, TE-10), especially in KYSE150 and TE-10. To sum up, we illustrated that SHMT2 expression was dramatically upregulated and related to the poor prognosis in EC.

### Knockdown of SHMT2 inhibits malignant development and immune escape in EC cells

**Fig. 2:**
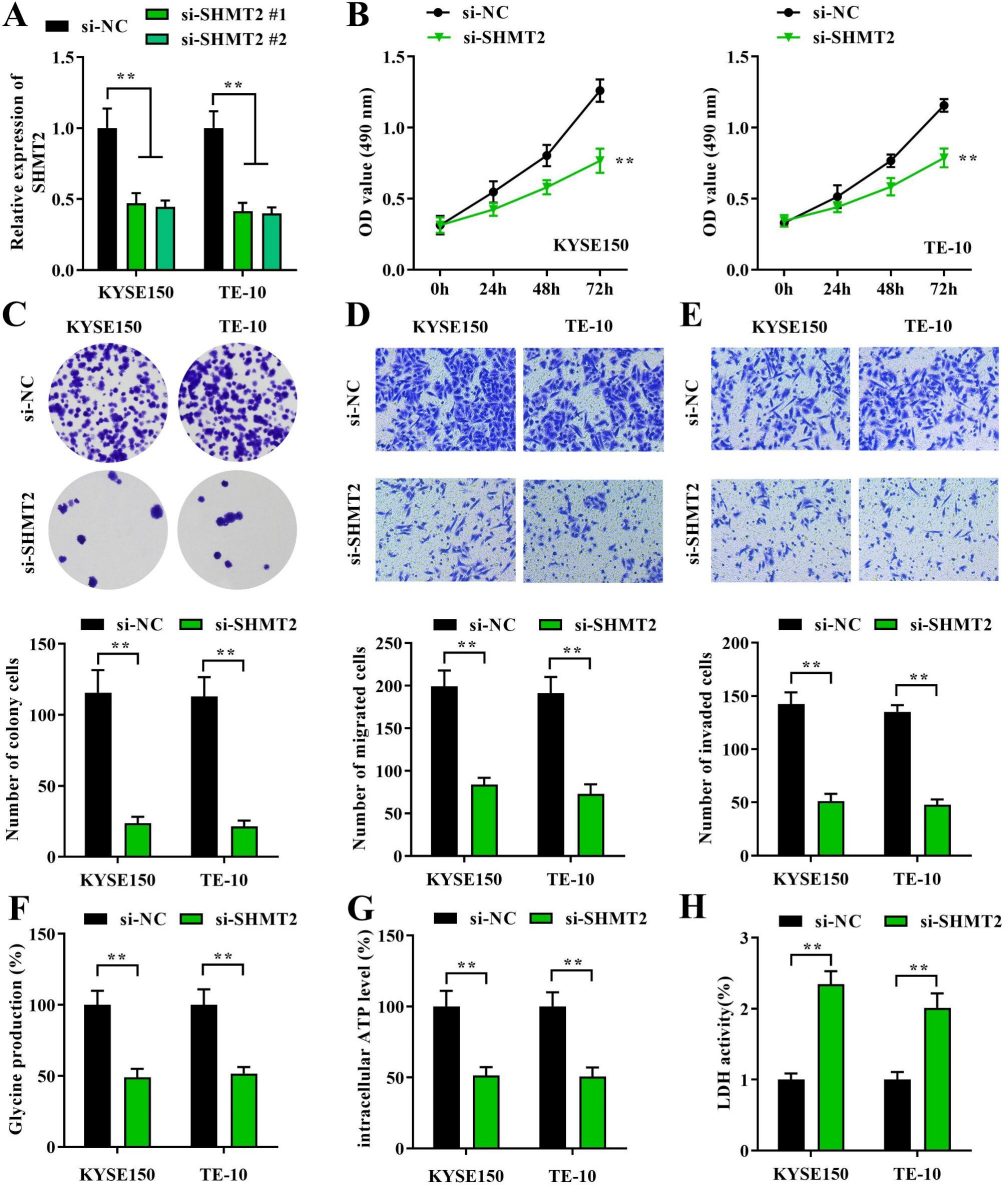
The effects of SHMT2 on EC development and glycolysis. **A**: knockdown transfection efficiency of SHMT2; **B**: CCK-8 assay was performed to assay cell viability; **C**: colony formation assay was employed to evaluate cell proliferation; **D-E**: Transwell assay was applied to analyze cell migration and invasion ability; **F-G**: kits were adopted to measure glycine production and ATP levels. H: The level of LDH after co-culture with T cells. *P < 0.05; **P < 0.01; ***P < 0.001

In recent years, the function of SHMT2 in tumors has received much attention. It has been demonstrated thatSHMT2 promotes tumor cell proliferation by converting serine to glycine [17]. Previously, our data confirmed that SHMT2 was highly expressed in EC. However, the specific function of SHMT2 in EC malignant progression still needs further clarification. To deal with this, we selected KYSE150 and TE-10 cells due to its high SHMT2 expression for siRNA transfection experiments. The knockdown efficiency of SHMT2 was first verified using RT-qPCR. We found that the SHMT2 was obviously reduced in the both of si-SHMT2#1 and si-SHMT2#2 transfected KYSE150 and TE-10 cells (Fig. 2A). Also, we chose si-SHMT2#2 with slightly higher knockdown efficiency for subsequent experiments, and labeled it as si-SHMT2. Next, the effects of SHMT2 knockdown on cell proliferation, migration and invasion were assessed by MTT assay, colony formation assay and transwell assay. The results revealed that knockdown of SHMT2 clearly limited the growth of KYSE150 and TE-10 cells (Fig. 2B). Cell transfected with si-SHMT2 also exhibited a decrease trend of clonogenic ability (Fig. 2C). These indicated that knockdown of SHMT2 remarkably restrained the proliferation ability of EC cells. Transwell assays showed that silencing SHMT2 sharply suppressed the migration and invasion ability of KYSE150 and TE-10 cells (Fig. 2D-E). In order to achieve high proliferation rate, tumor cells require a carbon unit for nucleotide synthesis, methylation, and reduction metabolism. Among them, the serine/glycine metabolic pathway has received increasing attention in recent years [18, 19]. Therefore, to investigate whether SHMT2 knockdown also affected on intracellular one carbon metabolism, we examined the glycine production and ATP level. We observed that the levels of glycine (Fig. 2F) and ATP (Fig. 2G) were evidently reduced in SHMT2 knockdown cells, indicating that intracellular glycine metabolism was limited. Eventually, we also tested whether SHMT2 participates in the immune response of EC. After co-culturing EC cells with T cells and we co cultured these two types of EC cells with T cells, we found that SHMT2 knockdown obviously induced the activity of LDH in KYSE150 and TE-10 cells, indicating that SHMT2 silencing enhanced the killing effect of T cells on EC cells (Fig. 2H). The above data together displayed that the SHMT2 silencing inhibited the proliferation, migration, and invasion of EC cells by suppressing cellular immune escape.

### SHMT2 regulates the level of c-myc through m6A modification

**Fig. 3:**
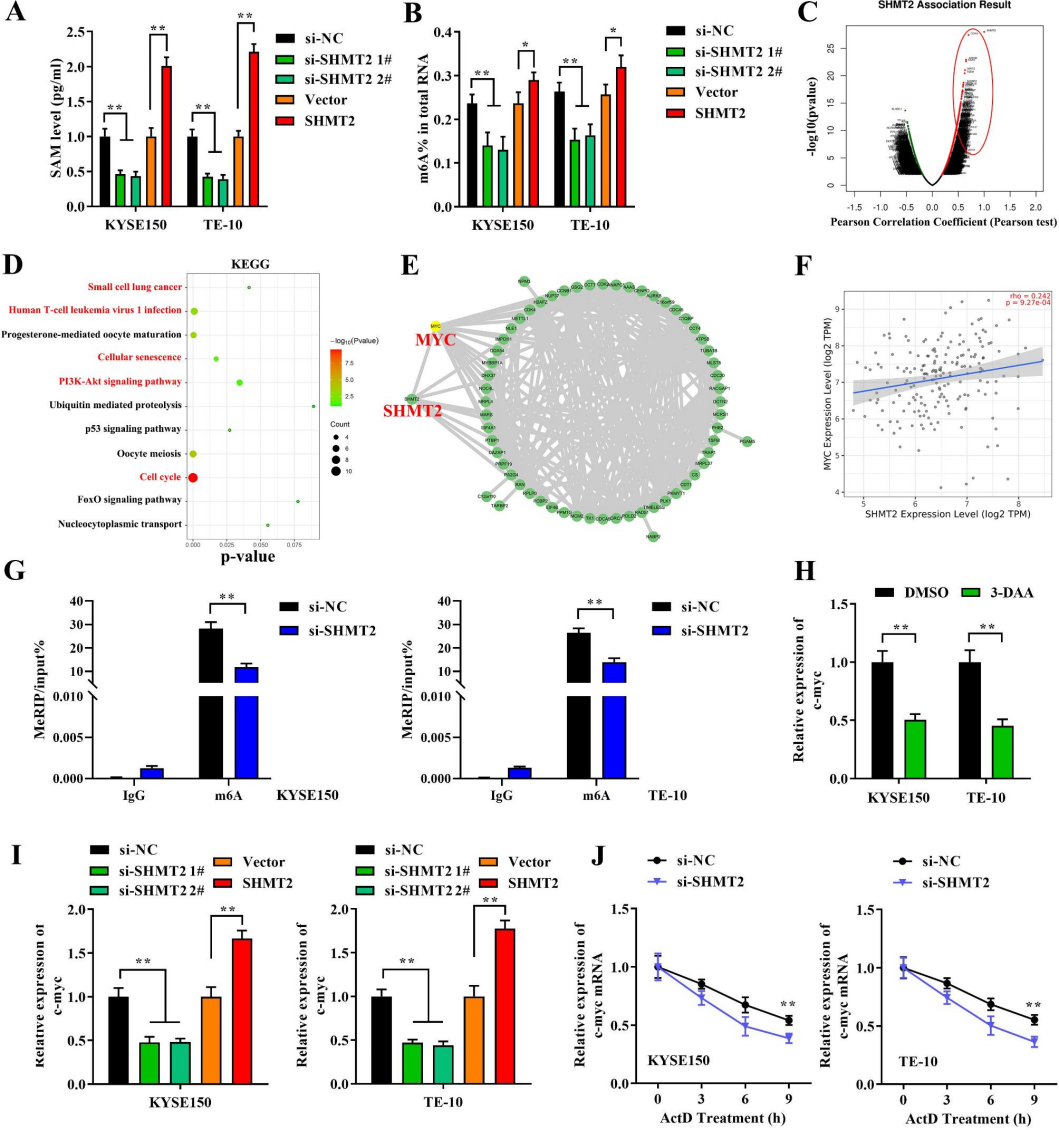
SHMT2 mediated m6A modification of c-myc. **A**: SAM levels; **B**: overall cellular m6A levels was measured after SHMT2 knockdown or overexpression; **C**: LinkedOmics (http://www.linkedomics.org/login.php) was applied to analyze the genes correlated withSHMT2; **D**: KEGG (R language) analysis was used to find enrichment pathways of positively genes correlated ( > = 0.05) with SHMT2; **E**: String (https://cn.string-db.org/cgi/input?sessionId=bsbz3beplR12&input_page _show_search = on) was performed to analyze the interaction of SHMT2 with these genes; **F**: the correlation between SHMT2 and MYC expression in EC; **G**: MeRIP was conducted to detect m6A levels of c-myc after knockdown of SHMT2; **H**: 3-DAA treatment; **I**: the mRNA levels of c-myc after knockdown and overexpression of SHMT2;** J**:Act D assay. *P < 0.05; **P < 0.01; ***P < 0.001

In eukaryotes, m6A methylation is the most abundant modification in mRNA and is involved in almost all stages of the RNA cycle, including mRNA transcription, maturation, translation, degradation and stability. Studies have revealed that m6A RNA methylation is widely involved in the metabolic reorganization of tumor cells. And changes of m6A also affect tumor progression, including proliferation, growth, invasion, and metastasis [20]. Therefore, to investigate whether SHMT2 played its biological function based on m6A modification in EC, we first examined the levels of SAM, an important intracellular methyl donor. Notably, knockdown of SHMT2 significantly suppressed intracellular SAM levels in KYSE150 and TE-10 cells. In contrast, overexpression of SHMT2 contributed to the accumulation of SAM in the cells (Fig. 3A). Next, quantification of intracellular m6A levels emerged the same trend. Specifically, knockdown or overexpression of intracellular SHMT2 expression clearly reduced or induced m6A levels, respectively (Fig. 3B). Subsequently, to further explore the downstream mechanisms of SHMT2, we analyzed genes which associated with SHMT2 using the LinkedOmics online tool. Figure 3C presented positively (red)/negatively (green) genes interacted with SHMT2 (Fig. 3C). KEGG analysis demonstrated the enrichment pathways of positively correlation genes of SHMT2 (Fig. 3D). Next, we analyzed the protein interaction network of SHMT2 linked genes by String database. The analysis indicated that MYC was the highest correspondence protein interactions of SHMT2 (Fig. 3E). In addition, MYC expression was analyzed based on TCGA database. MYC expression was significantly upregulated in EC (fig. S1A). We also found that MYC was positively correlated with the clinical stage of EC. Specifically, MYC in tumor tissues with high TNM stage (III-V) was clearly higher than that in patients with low stage (I-II) (fig. S1B). To further confirm the relationship between SHMT2 and MYC in EC, we analyzed the expression correlation of SHMT2 and MYC. Unexpectedly, the expression of SHMT2 in EC displayed a clearly positive correlation with MYC expression (Fig. 3F). However, whether SHMT2 mediates the m6A modification of c-myc still needs further study. By MeRIP assay, we found that SHMT2 knockdown obviously limited m6A levels of c-myc in KYSE150 and TE-10 cells (Fig. 3G). To verify whether m6A methylation affects c-myc expression, we treated cells with 3-DAA (a global inhibitor of SAM synthesis) and DMSO was served as a control treatment. We subsequently observed that the c-myc mRNA was remarkably decreased in 3-DAA-treated KYSE150 and TE-10 cells (Fig. 3H). In addition, whether SHMT2 affects the expression of c-myc in KYSE150 and TE-10 cells was further verified. Knockdown or overexpression of SHMT2 was able to markedly weak or strengthen the expression of c-myc mRNA, respectively (Fig. 3I). Finally, the effect of SHMT2 on c-myc mRNA stability was examined by ActD assay. Knockdown of SHMT2 distinctly repressed the half-life time of c-myc mRNA thus accelerating the degradation of c-myc mRNA (Fig. 3J). In conclusion, SHMT2 regulated c-myc expression and maintained its mRNA stability in EC cells through mediating m6A modification.

### METTL3/FTO/ALKBH5 mediates m6A modification of c-myc

**Fig. 4:**
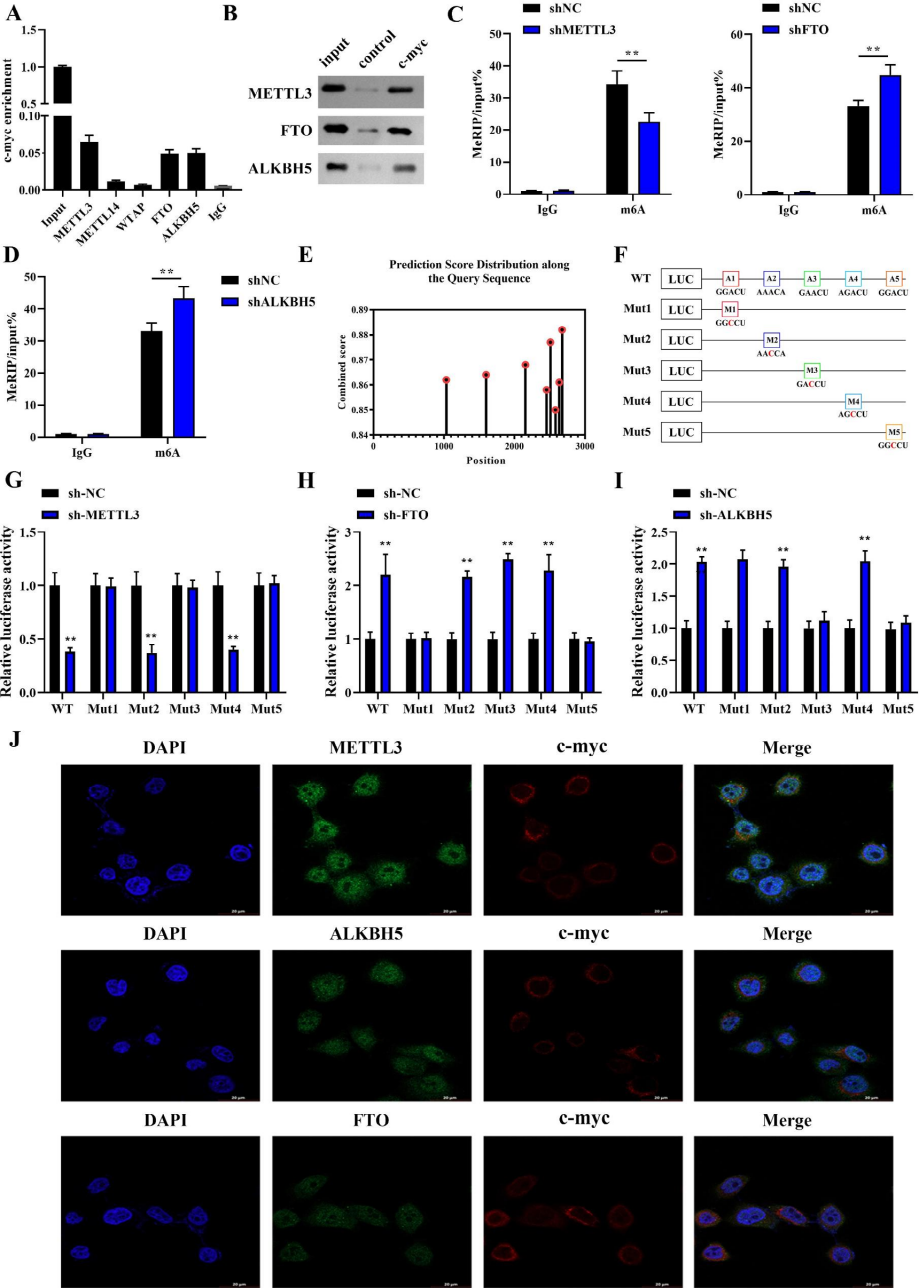
METTL3/FTO/ALKBH5 regulated the m6A modification of c-myc. **A**: RIP assay was performed to check the interaction effect between MYC and methylation transferase as well as demethylase; **B**: RNA pull-down was conducted to detect interaction effects between MYC and METTL3, FTO or ALKBH5; **C-D** m6A levels of MYC was measured after knockdown of METTL3, FTO, and ALKBH5; **E**: SRAMP analysis (http://www.cuilab.cn/sramp) was applied to predict m6A modification sites on MYC mRNA; **F**: schematic diagram of m6A site mutation construct; **G-I**: dual luciferase reporter vector assay was employed to confirm the modification sites of METTL3/FTO/ALKBH5 on MYC; **J**: immunofluorescence in situ hybridization was conducted to verify the co-localization of METTL3/FTO/ALKBH5 and MYC in cells Situation. *P < 0.05; **P < 0.01; ***P < 0.001

The modification of m6A is dynamic and reversible, and its biological role is mainly regulated by “writers”, “erasers” and “readers”. m6A modification deposition is mainly catalyzed by the multi-component methyltransferase complex consisting of METTL3, METTL14 and WTAP, which are the earliest known m6A-writers. While m6A-erasers, such as FTO and ALKBH5, mainly perform the reversible step of m6A methylation by demethylation [21]. Therefore, to explore the regulatory mechanism of m6A modification of c-myc, RIP and RNA pull down experiments were performed to analyze the interaction effects between c-myc and methylation transferases as well as demethylases. We found that the methylation transferase METTL3 and the demethylases FTO and ALKBH5 were able to significantly enrich MYC (Fig. 4A and B). TCGA database results showed that METTL3, ALKBH5 and FTO were upregulated with different degrees in EC (fig. S2A). In addition, we only found that METTL3 was positively correlated with the clinical stage of EC, while ALKBH5 and FTO were not (fig. S2B). Moreover, METTL3 silencing in cells notably suppressed c-myc expression (fig. S2C). Consistent with this, when we treated the cells with STM2457, a selective METTL3 inhibitor, the expression of c-myc was also decreased (fig. S2D). Next, the m6A modification of MYC regulated by METTL3, FTO and ALKBH5 was further verified using MeRIP. We examined the m6A levels of MYC after METTL3, FTO or ALKBH5 knockdown in cells. We revealed that the m6A level of MYC was obviously reduced when METTL3 was suppressed (Fig. 4C). In contrast, knockdown of FTO and ALKBH5 remarkably induced the m6A level of MYC (Fig. 4C-D). Figure 4E displayed the m6A modification sites on c-myc mRNA sequences predicted by SRAMP online tool. We then selected the five highest confidence m6A sites for mutation construction (Fig. 4F). Subsequently, a dual luciferase reporter vector was employed to check. Interestingly, METTL mediated the m6A modification of MYC through sites 1,3,5 (Fig. 4G). FTO mediated the m6A modification of MYC through sites 1, 5 (Fig. 4H). ALKBH5 mediated the m6A modification of MYC through sites 3,5 (Fig. 4I). Finally, immunofluorescence in situ hybridization analysis confirmed that all of the METTL3/FTO/ALKBH5 co-localized with MYC in cells (Fig. 4J). Finally, we mapped SHMT2, MYC, and m6A regulator (METTL3, FTO, and ALKBH5) protein interactions using STRING software (fig. S2E). Overall, SHMT2-regulated m6A modification of MYC was evidenced to be mediated by the methylation transferase METTL3 and the demethylation enzymes FTO and ALKBH5.

### IGF2BP2 regulates the m6A modification of c-myc

**Fig. 5:**
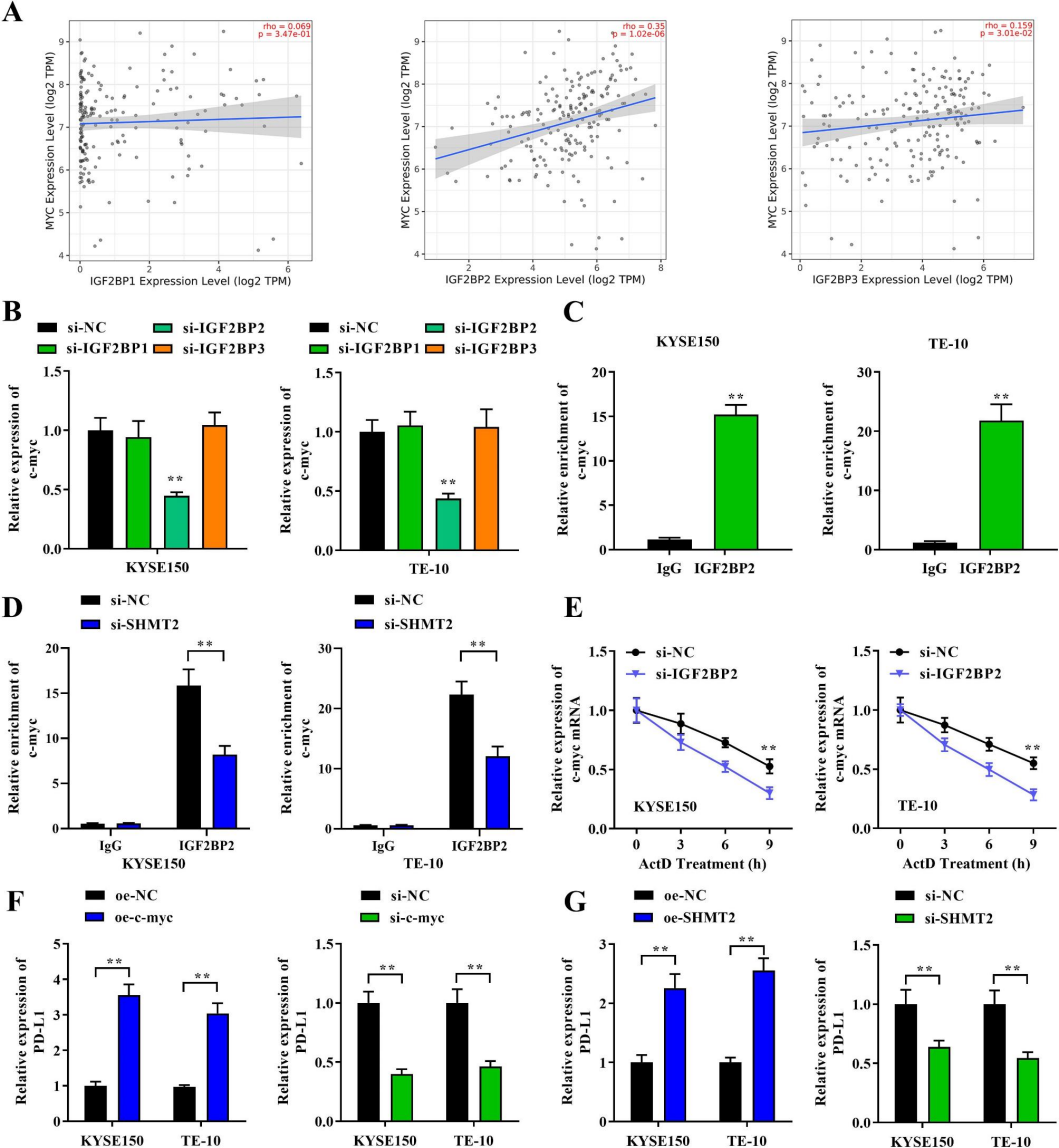
IGF2BP2 mediated c-myc m6A modification. **A**: Analysis of the correlation between IGF2BP1/2/3 and cmyc expression in EC; **B**: RT-qPCR was employed to analyze c-myc mRNA expression after knockdown of IGF2BP1/2/3; **C**: RIP assay was applied to verify the binding between IGF2BP2 and c-myc; D: SHMT2 affect the m6A modification of c-myc via IGF2BP2; E: Act D treatment; **F**: The expression of PD-L1 was detected after MYC overexpression or knockdown; **G**: The expression of PD-L1 was detected after SHMT2 overexpression or knockdown. *P < 0.05; **P < 0.01; ***P < 0.001

The aberrant of m6A in cancer mainly depend on the expression and activity of “writers” and “erasers”; whereas the recognition and regulation of target mRNA m6A modifications are mainly determined by “readers”. As one of the most prominent m6A reader families, IGF2BP family of “readers” are responsible for recruiting RNA stabilizers to promote mRNA stabilization and thus influence tumor progression [22]. Therefore, we continued to explore the regulatory mechanisms of m6A modifications in MYC in depth. We first analyzed the correlation between IGF2BP family members IGF2BP1, IGF2BP2 and IGF2BP3 and MYC expression in EC. The results displayed that MYC expression showed obvious positive correlation with IGF2BP2 or IGF2BP3 expression in EC (Fig. 5A). To further verify the functions of IGF2BP2 and IGF2BP3, c-myc expression was detected after IGF2BP2 and IGF2BP3 knockdown in cells. Notably, a significant decrease of c-myc expression was only observed in KYSE150 and TE-10 with IGF2BP2 knockdown (Fig. 5B). The TCGA database results showed that IGF2BP2 was induced in EC (fig. S3A). In addition, IGF2BP2 was also positively correlated with the clinical stage of EC (fig. S3B). Next, we verified the binding relationship between IGF2BP2 and c-myc by RIP assay. In KYSE150 and TE-10 cells, c-myc was remarkably enriched by IGF2BP2 (Fig. 5C). However, in SHMT2 knockdown cells, the enrichment of c-myc was clearly limited (Fig. 5D). In addition to this, knockdown of IGF2BP2 similarly restrained the half-life of c-myc mRNA (Fig. 5E). The above results demonstrated that SHMT2 mediated the m6A modification and maintained stability of c-myc through IGF2BP2. Finally, to explore the targets of SHMT2 and MYC on immune escape in tumor cells, we also examined PD-L1 expression. We found that overexpression of SHMT2 or MYC promoted PD-L1 expression in EC cells. In contrast, knockdown of SHMT2 or MYC expression suppressed PD-L1 expression (Fig. 5F and G). This suggested that SHMT2 regulated PD-L1 expression in cells through MYC, thereby modulating the immune escape of EC.

### SHMT2 mediated c-myc m6A modification through METTL3 to regulate malignant development and immune escape in EC cells

**Fig. 6:**
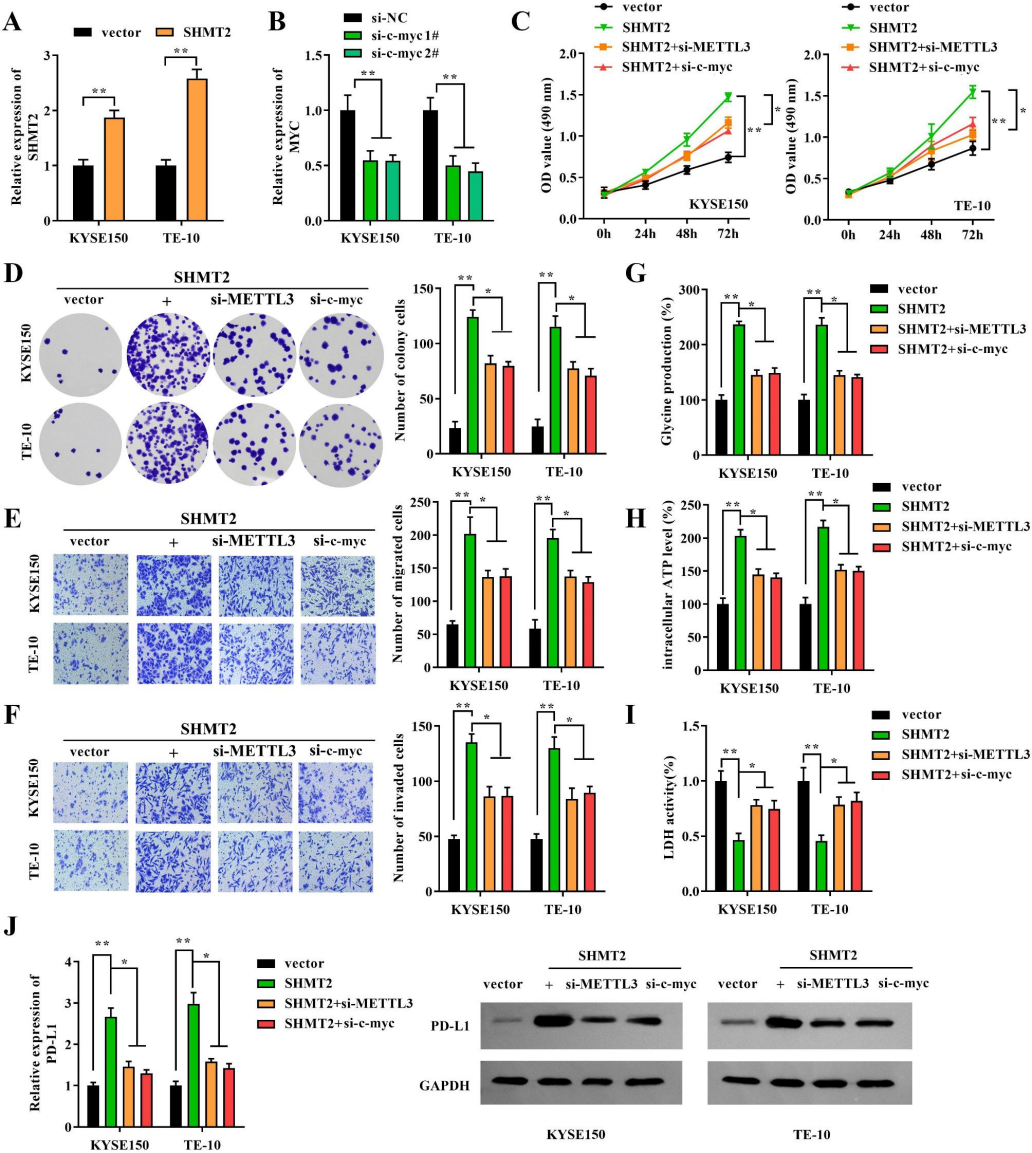
SHMT2 regulated EC progression and immune escape by mediating m6A of c-myc. **A-B**: RT-qPCR was conducted to detect transfection efficiency; **C**: CCK-8 was performed to examine cell viability; **D**: colony formation assay was applied to evaluate cell proliferation; **E-F**: Transwell was employed to measure cell migration and invasion ability; **G-H**: kits was used to detect glycine production and ATP levels. **I**: The level of LDH after co-culture with T cells; **J**: PD-L1 expression was examined. *P < 0.05; **P < 0.01; ***P < 0.001

Previously, we demonstrated that SHMT2 regulated m6A modification of c-myc through METTL3-FTO/ALKBH5-IGF2BP2 regulators. Therefore, to further demonstrate whether SHMT2 regulates malignant progression and immune escape of EC cells through this mechanism, rescue experiments were designed to verify. First, the transfection efficiency of SHMT2 vector and si-c-myc was examined by RT-qRCR. We observed that SHMT2 vector or si-c-myc transfection obviously induced SHMT2 (Fig. 6A) or reduced the expression of c-myc in KYSE150 and TE-10 cells (Fig. 6B), respectively. Similarly, we chose si-MYC#2 with slightly higher knockdown efficiency for subsequent experiments, and labeled it as si-MYC. Next, cell function assays were performed to examine cell proliferation, migration, invasion and immune escape capacity. Clearly, overexpression of SHMT2 significantly augmented the proliferative capacity of the cells. However, knockdown of METTL3 or c-myc significantly limited the proliferation ability of the cells (Fig. 6C-D). Similarly, the migration and invasion ability of the cells exhibited the same changes of trend (Fig. 6E-F). In addition, overexpression of SHMT2 also enhanced intracellular glycine metabolism, as evidenced by remarkably increase of glycine and ATP production. In contrast, knockdown of METTL3 or c-myc also re-wreaked glycine and ATP levels in cells (Fig. 6G-H). Furthermore, LDH activity was decreased in SHMT2 overexpression cells. While it was re-strengthened after knockdown of METTL3 or c-myc (Fig. 6I). Finally, the promotion of PD-L1 by SHMT2 overexpression was largely eliminated by METTL3 or c-myc silencing (Fig. 6J). These data supported that SHMT2 affected proliferation, migration, invasion and immune escape of EC cells via c-myc m6A modification in a METTL3 dependent manner.

### Knockdown of SHMT2 inhibits Tumor growth in vivo by suppressing the m6A level of MYC

**Fig. 7:**
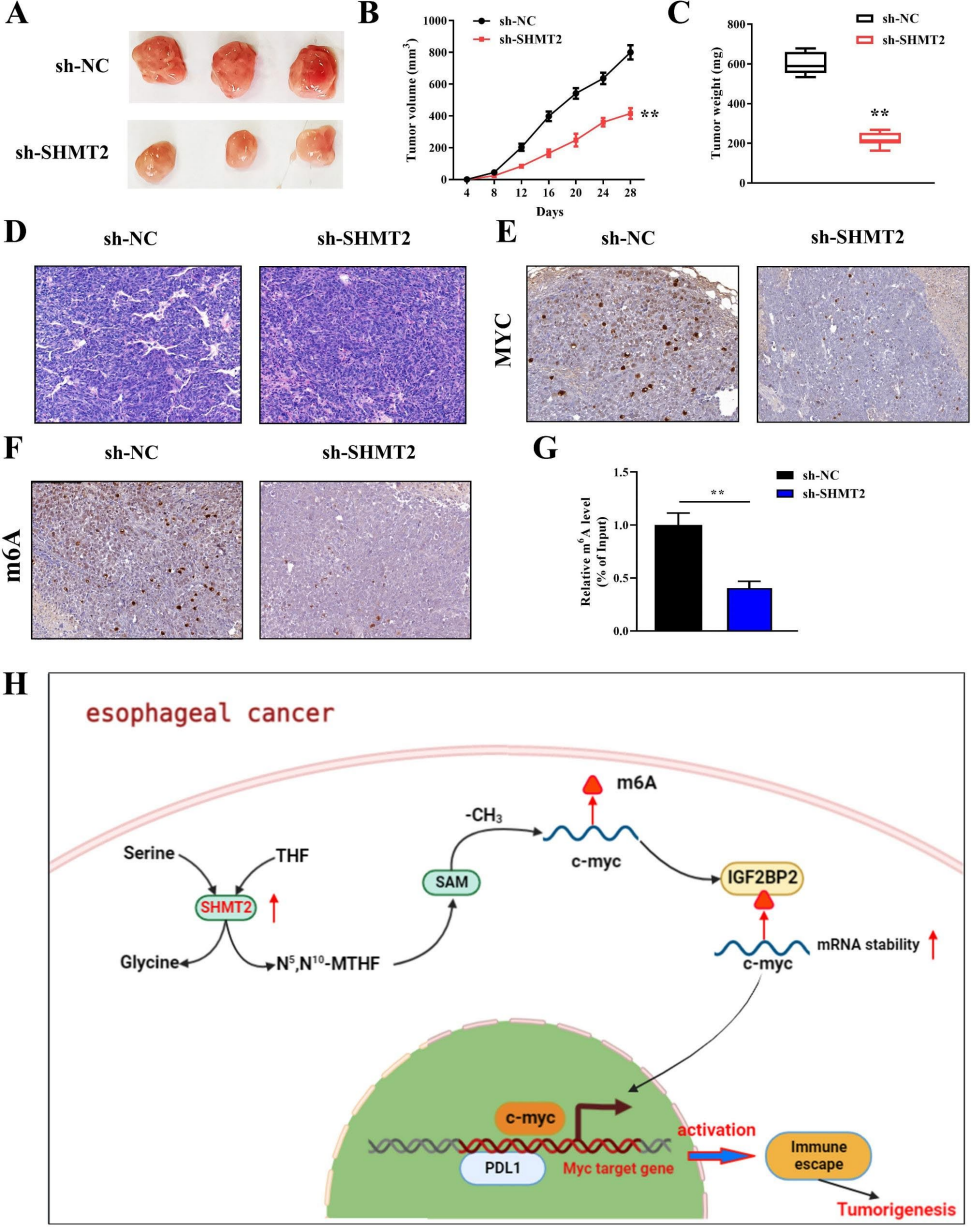
Role of SHMT2 on tumor growth in vivo. **A**: images of subcutaneous tumors; **B**: tumor growth curves; **C**: tumor weights in nude mice; **D**: images of HE staining; **E**: immunohistochemistry was conducted to analyze MYC expression in tissues; **F**: immunohistochemistry was employed to evaluate m6A levels in tissues; **G**: m6A levels of MYC was measured by MeRIP; **H**: mechanistic hypothesis map. *P < 0.05; **P < 0.01; ***P < 0.001

Finally, a xenograft tumor nude mouse model was conducted to verify the role and mechanism of SHMT2 in vivo. Unexpectedly, knockdown of SHMT2 obviously slowed down tumor growth in nude mice, as evidenced by smaller tumor size and lighter weight (Fig. 7A-C). Subsequently, tumor tissues were collected for HE staining and immunohistochemical analysis. Figure 7D displayed the representative images of HE staining. The results showed that the sh-NC group had diverse cell shapes, irregular arrangement, high nuclear to cytoplasmic ratio, and a large number of nuclear divisions in transplanted tumor tissue, which were typical characteristics of tumor tissue. Notably, both MYC expression and m6A levels of nude mice tumor tissues were reduced in the SHMT2 knockdown group (Fig. 7E-F). Consistent with this, SHMT2 silencing remarkably suppressed the m6A levels of MYC in nude mice tumor tissues (Fig. 7G). In conclusion, these data containing *in vivo and in vitro* revealed that SHMT2 promoted the accumulation of methyl donor SAM through one carbon metabolism pathway, which mediated MYC expression in an m6A-dependent manner, thereby activating EC immune escape and tumorigenesis (Fig. 7H).

## Discussion

EC is generally classified into two subtypes, esophageal squamous cell carcinoma (ESCC) and esophageal adenocarcinoma (EAC). ESCC is a multifactorial disease. External factors are related to include tobacco, alcohol and dietary habits, while internal factors are attributed to molecular events [23]. However, the precise molecular events related to the pathogenesis of EC have not been fully elucidated, which has led to limited targeted therapy and inadequate clinical management of patients with EC. Therefore, further study of esophageal carcinogenesis and progression detailed mechanisms could help to find and identify potential therapeutic markers and thus improve the prognosis and treatment of EC patients.

In the past few years, cellular metabolic reprogramming, representing cancer-related metabolic changes during tumorigenesis, has been recognized as a hallmark of human cancer and plays a key role in human cancer cell proliferation and survival. Tumor cells reprogram their metabolism to support cell growth, proliferation and differentiation, thereby driving cancer progression [24]. Serum metabolomic analysis identified significant changes in lipid metabolism, amino acid metabolism, glycolysis, ketogenesis, tricarboxylic acid (TCA) cycle and energy metabolism of EC patients [25]. Serine/glycine, one-carbon unit, and folate metabolism have been the hot topics of research in tumor metabolism in recent years. Recent studies on cancer metabolomics have shown that glycine metabolism is associated with cancer cell proliferation. Phosphoserine aminotransferase (PSAT) and serine hydroxymethyltransferase (SHMT) linked to the serine and glycine catabolism can induce tumor formation in vivo [17]. SHMT2 is one of the notable metabolic enzymes that plays a key role in the conversion of serine to glycine. It reported that SHMT2 is upregulated and associated with tumor progression in a variety of cancers [26, 27]. For example, Sara et al. revealed that SHMT2 inhibition or impairment in lymphoma affected S-adenosylmethionine synthesis, which induced alterations in DNA and histone methylation, and was synergistically amplified with bcl2 thereby promoting lymphatic damage [28]. SHMT2 was aberrantly highly expressed in head and neck cancer. It regulated tumor progression by participating in cancer cell stemness [29]. SHMT2 not only enhanced tumor proliferation, but also associated with immunity. Wu et al. illustrated that high SHMT2 expression was associated with advanced pathological grading and recurrence of oral squamous cell carcinoma [30]. However, the potential functions and mechanisms of SHMT2 in the malignant progression of EC still need to be explored in depth.

Here, we also identified the involvement of SHMT2 in EC progression, which may provide a basis for further therapies targeting the SHMT2 pathway in the clinic. Specifically, our analysis revealed an abnormal upregulation of SHMT2 expression in EC using TCGA and HPA data. Clinical data also suggested that SHMT2 expression is abnormally upregulated in EC and correlates with prognosis. Through functional assay analysis, we observed that knockdown of SHMT2 inhibited the proliferation, migration and invasive ability of EC cells. Metabolic reprogramming is one of the main features of malignant tumors and facilitates tumor progression. To meet the demands of rapid generation, cancer cells can acquire metabolic adaptations through multiple endogenous and exogenous signaling pathways, providing a large amount of energy for the uninterrupted replication process [31]. Therefore, to further explore whether SHMT2 regulates this metabolic alteration in EC, we examined intracellular glycine and ATP production. We found that knockdown of SHMT2 inhibited the production of glycine and ATP, indicating that glycine was limited. Moreover, we also measured the link between SHMT2 and tumor immune. SHMT2 was found to enhance T cell mediated tumor killing effect. PD-L1, a tumor cell surface transmembrane protein that is commonly highly expressed in a variety of cancers. Studies have shown that PD-L1 can bind to PD-1 on T cells and activate the downstream signaling of the PD-1 receptor in T cells, thereby inhibiting T cell responses by decreasing T cell activity and accelerating their apoptosis, thus protecting tumor cells from immune attack and causing tumor immune escape [32]. Immunotherapy targeting the PD-1/PD-L1 axis has also been shown significant antitumor effects [33]. Therefore, PD-L1 in tumor cells is considered as an important tumor immune target. By detecting PD-L1 expression in EC cells, we found that SHMT2 overexpression promoted PD-L1 expression. In contrast, knockdown of SHMT2 suppressed PD-L1 expression. Taken together, these data demonstrated that SHMT2 may regulate immune escape pathway through one-carbon metabolism, which in turn promoted the malignant progression of EC.

Furthermore, cellular metabolome and epigenome interact in a bidirectional manner, and regulated genetic and molecular drivers of cancer. Epigenetic abnormalities regulate the expression of many metabolic genes and thus play an important role in metabolic reorganization and redox homeostasis in cancer cells [34]. N6-methyladenosine (m6A) methylation, the most abundant and prevalent mRNA modification in eukaryotic cells, controls various aspects of modified transcripts and has been implicated in carcinogenesis and tumor progression [35]. Recently, several key studies demonstrated that alterations in core genes during m6A modification affect tumorigenesis, cancer cell proliferation, tumor microenvironment, and cancer prognosis in a variety of human cancers, including EC [36–38]. Therefore, targeting the m6A locus has emerged as a promising cancer therapeutic strategy to increase clinical benefit and needs to be further explored in the future. Similar to other epigenetic modifications, m6A controlled RNA fate and gene expression at the post-transcriptional level. For example, Ge et al. showed that METTL3 affects the malignant biological behaviors of EC such as proliferation, migration, invasion, and the immune microenvironment of the tumor by regulating IFIT2 [39]. Zhao et al. demonstrated that there is a synergistic relationship between FTO and ERBB2, specifically FTO regulates tumorigenesis and metastasis of EC through mediated ERBB2 m6A modification [40]. Han et al. similarly revealed that METTL3 promotes the expression of NOTCH1 and activates the Notch signaling pathway through mediated m6A modification to promote the development of EC [41]. All these studies indicate that m6A methylation is involved in regulating the malignant phenotype of tumors by controlling the expression of tumor-related genes, and that aberrant m6A methylation levels promote tumor development. The development of applicable inhibitors, such as m6A enzyme inhibitors or modified RNA methylation inhibitors, may point to a new direction in cancer treatment and should be further explored in cancer therapy. However, due to the complex regulatory mechanisms, the clear roles of m6A and its regulatory proteins in cancers are still limited, and the mechanism has not been well characterized, such as EC. Thus, screening and identification of downstream targets are still the core on m6A modification in EC, and more drugs and new therapeutic strategies related to m6A remain to be explored.

SAM is a universal methyl donor that is utilized by methyltransferases to methylate DNA, RNA, metabolites and proteins. Methylation modifications are dynamic and tightly regulated. Since SAM concentration fluctuates in cells and affects methyltransferase activity, SAM levels affect histone methylation levels [42]. Therefore, to further investigate whether SHMT2 functions in an m6A-dependent manner in EC, we assessed intracellular SAM and m6A levels. Interestingly, knockdown or overexpression of SHMT2 suppressed or increased SAM and m6A levels. This suggested that the role of SHMT2 in EC was m6A modification-dependent manner. Metabolic enzymes play a crucial role in metabolic regulation. Metabolic enzymes directly or indirectly alter gene and protein expression of oncogenes in response to intra/extracellular signaling of metabolic demands of cancer cells, thus affecting cancer progression [43, 44]. Therefore, we searched for proteins that interact with SHMT2 through bioinformatic analysis of online tools. MYC was the most significantly associated protein with SHMT2 that we analyzed. SHMT2 was positively correlated and regulated MYC expression. In addition, knockdown of SHMT2 was able to reduce the level of m6A modification of c-myc.

MYC, an important family of transcription factors including c-MYC, n-MYC and I-MYC, is essential for cell growth, metabolism and development. Members of the MYC family are currently the most important transcription factors and widely available nuclear oncogenes. MYC is also recognized as an important poor prognostic factor in EC, and MYC overexpression promotes malignant progression of EC cells [45]. Similar results were obtained in our study, where knockdown of MYC partially eliminated the promotional effects of SHMT2 overexpression on EC cell proliferation, migration, invasion and immune escape. c-Myc plays an important role in regulating cancer metabolism as well. For example, Under stress conditions of nutritional deficiencies such as glucose or glutamine, tumor cells support survival by regulating the expression of metabolic enzymes and activating the oncogene c-Myc [31]. In colorectal cancer, GLCC1 stabilized the transcription factor c-Myc by regulating the ubiquitination of c-myc, which further regulated the transcriptional modifications of downstream genes and induced cancer cell proliferation and survival by enhancing glycolysis [46]. In addition, there was a close link between c-myc and epigenetic factors. m6A regulators, such as methylesterases (e.g., METTL3), demethylases (e.g., ALKBH5) and readers (e.g., IGF2BP2), regulate the m6A modification and stability of c-myc by regulating the expression of multiple signaling pathways and molecules. Several studies demonstrated that c-myc affected tumor progression in an m6A-dependent manner [47–50]. For example, in oral squamous cell carcinoma tumors, METTL3 enhances c-Myc stability and promotes cell progression through YTHDF1-mediated m 6 A modification [41]. In gastric cancer, FOXA2-mediated FTO stabilizes MYC mRNA by reducing m6A methylation of MYC. HDAC3 initiates tumor activity by regulating the FOXA2-mediated FTO/m6A/MYC axis [42]. In leukemia, METTL14, which is negatively regulated by SPI1, exerts its oncogenic effects by regulating m6A modification of its mRNA targets (e.g., MYB and MYC) [43]. In cervical cancer, HPV E6/E7 can regulate aerobic glycolysis in cells through IGF2BP2-mediated m6A-MYC mRNA stabilization, thereby promoting cancer progression [44]. The long non-coding RNA LINRIS boosted glycolysis and malignant progression in colorectal cancer through the IGF2BP2-c-Myc axis [51]. In lung adenocarcinoma, Wnt/β-catenin raised c-myc m6A levels via limiting FTO expression, thereby enhancing tumor glycolysis and tumorigenesis [52]. These studies suggest that targeting upstream and downstream of MYC m6A modifications may play a critical role in the mechanisms of cancer development and progression, which may represent a promising therapeutic modality. However, there is still a paucity of studies on the upstream and downstream of m6A modification targets of MYC, especially in EC, and the specific mechanisms still need to be intensified.

However, in EC, the mechanism by which m6A modulators regulated c-myc signaling remains largely unknown. Currently, only one relevant study was found showing that LINC00858 upregulates FTO expression by recruiting ZNF184 to inhibit m6A modification of MYC, which promotes proliferation, migration and invasive properties and accelerates apoptosis in EC cells [53]. And we found that knockdown of SHMT2 could reduce the m6A modification level of c-myc. In the present study, our results emphasized the regulatory role of SHMT2 on MYC through m6A modification and may provide clues for the development of therapeutic strategies for EC by targeting m6A modification and its related targets. In further mechanistic studies, we illustrated that the methylation transferase METTL3, demethylases FTO and ALKBH5, and the reading protein IGF2BP2 were involved in the m6A modification of c-myc. Mechanistically, SHMT2 augmented the accumulation of the methyl donor SAM through a serine/glycine single-carbon metabolic network, which contributed to maintain the mRNA stability and regulate m6A modification of c-myc in a METTL3/ FTO/ALKBH5/IGF2BP2-dependent manner. Rescue experiments confirmed that SHMT2 mediated c-myc m6A methylation through METTL3 to regulate proliferation, migration, invasion and immune escape of EC cells. In addition, in vivo experiments also revealed that SHMT2 accelerated EC tumorigenesis by MYC expression in an m6A-dependent manner. However, given that cellular metabolism is a complex process involving the activity of numerous genes, we cannot exclude that SHMT2 may also target tumor immunity and tumor progression by affecting the methylation status of mRNAs other than MYC in an m6A-dependent manner.

In recent years, targeting enzymes of one-carbon metabolism has emerged as a novel antitumor therapeutic strategy. However, antitumor therapy targeting one-carbon metabolic pathways still has many issues that need to be addressed. For instance, the clinical effectiveness of common clinical agents including pemetrexed, 5-fluorouracil, and methotrexate is limited by side effects. In addition, MTHFD1 can be activated compensatory after inhibition of MTHFD2 [54]. While co-inhibitors of SHMT1 and SHMT2, such as SHIN1, can be rapidly cleared in organisms without effective use [55]. Therefore, therapeutic strategies targeting one-carbon metabolism-related pathway and molecules still need more research in the future.

## Conclusion

In the present study, we confirmed the abnormal upregulation of SHMT2 expression in EC and it associated with poor prognosis by database analysis and clinical data. In vitro experiments showed that SHMT2 regulated c-myc expression in an m6A-dependent manner, thereby regulating cell proliferation, migration, invasion and immune escape. In vivo experiments revealed that SHMT2 promoted tumorigenesis by mediating m6A level of MYC. In conclusion, our data indicated that SHMT2 regulated malignant progression and immune escape of EC cells through c-myc m6A modification (Fig. 7H). This study elucidated a novel immune escape regulatory network that links the interactions between molecular drivers, metabolic reprogramming, and epigenetic modifications in EC, potentially providing new insights into the oncogenic mechanisms of EC, as well as promising approaches for treating patients with EC.

### Electronic supplementary material

Below is the link to the electronic supplementary material.

Supplementary Material 1

Supplementary Material 2

Supplementary Material 3

## Data Availability

The data that support the findings of this study are available from the corresponding author upon reasonable request.
