# Supplementary figures and images for: SHMT2 regulates esophageal cancer cell progression and immune Escape by mediating m6A modification of c-myc
Source: Cell Biosci. 2023 Nov 6;13:203. doi: 10.1186/s13578-023-01148-7 (PMC10629073; doi:10.1186/s13578-023-01148-7)

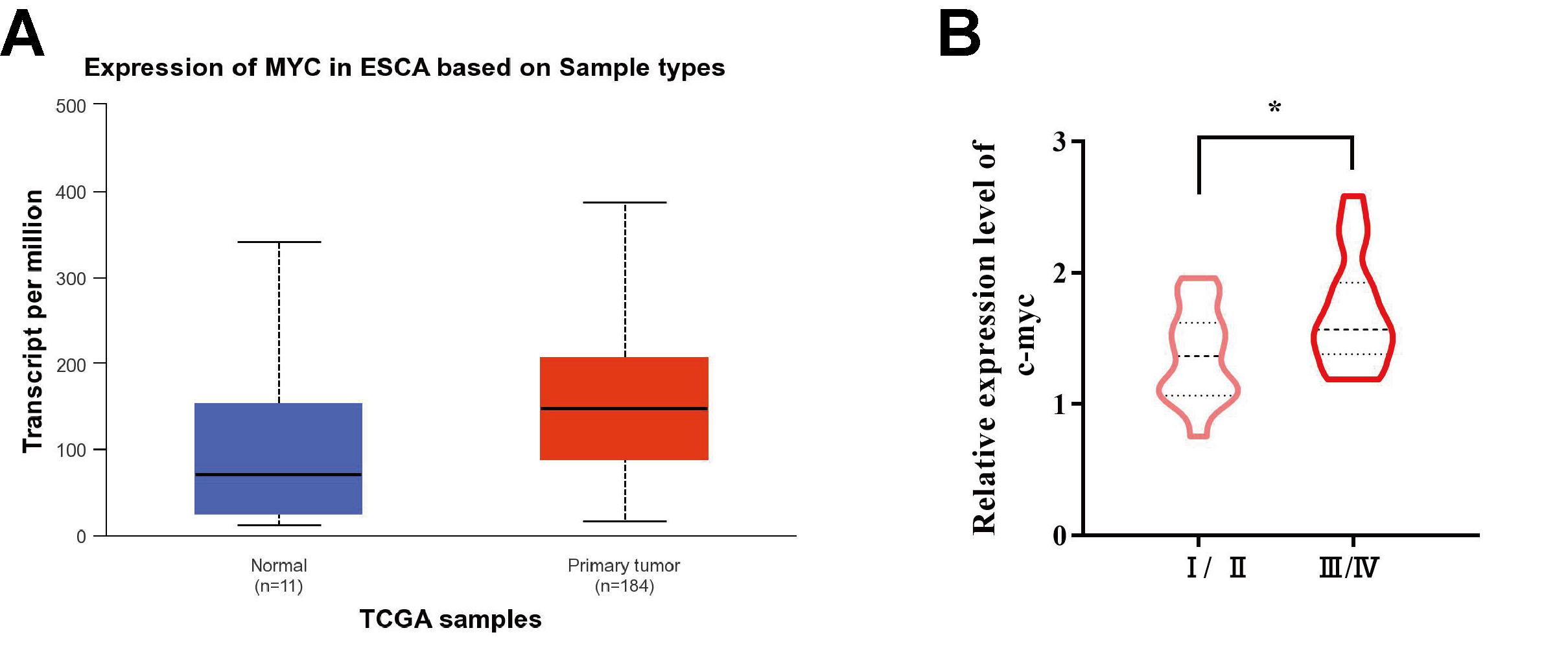

Supplement: Supplementary file 1 — Supplementary Material 1 [file 13578_2023_1148_MOESM1_ESM.tif]

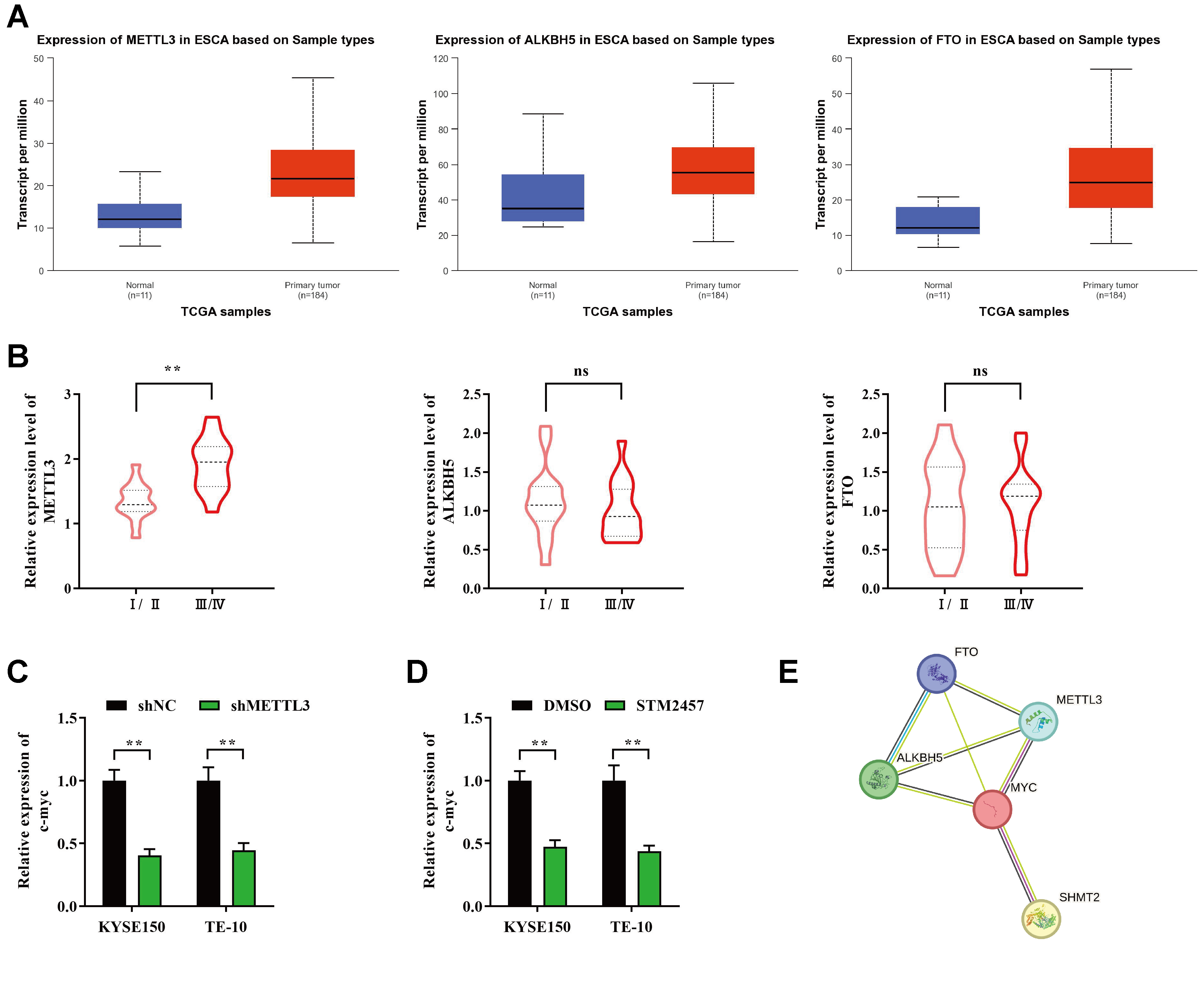

Supplement: Supplementary file 2 — Supplementary Material 2 [file 13578_2023_1148_MOESM2_ESM.tif]

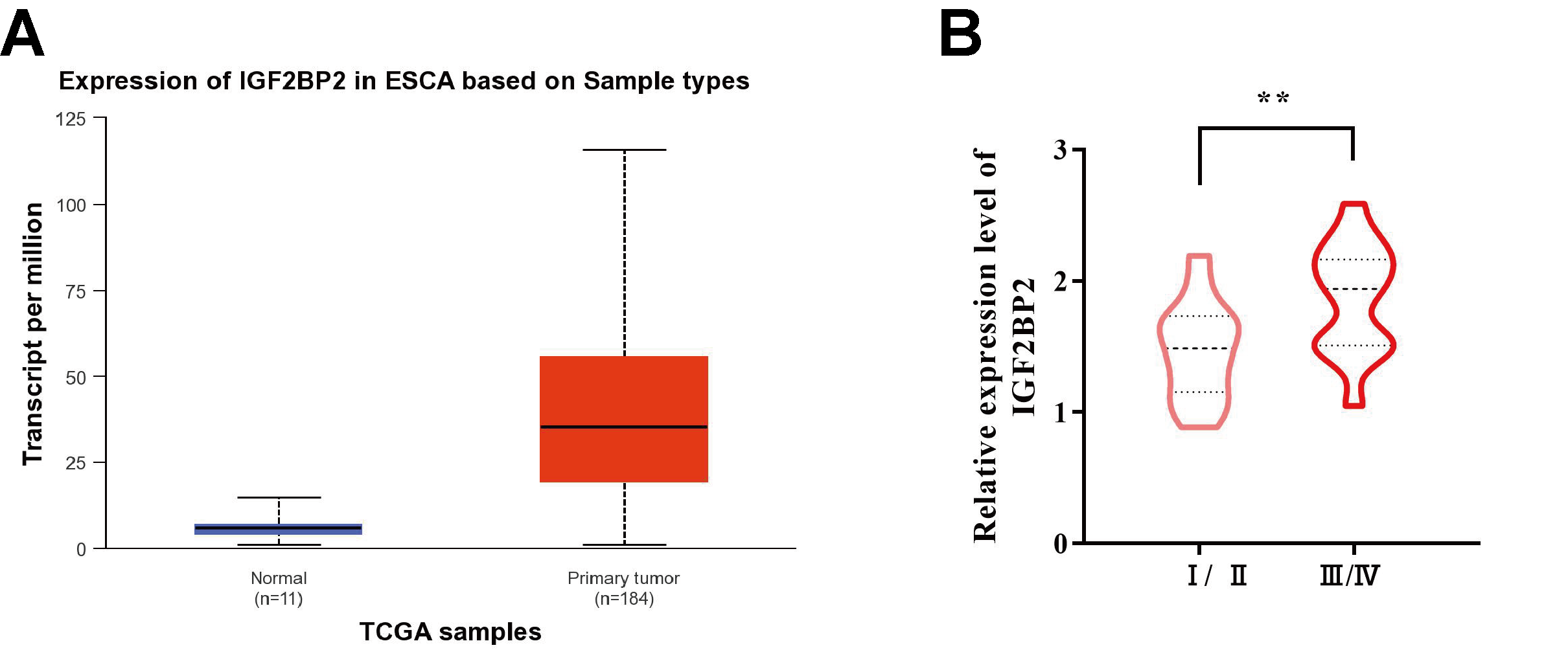

Supplement: Supplementary file 3 — Supplementary Material 3 [file 13578_2023_1148_MOESM3_ESM.tif]
